# Description of *Anoplophora fanjingensis* sp. n. (Coleoptera, Cerambycidae, Lamiinae) from southwest China

**DOI:** 10.3897/BDJ.8.e51752

**Published:** 2020-10-13

**Authors:** Shulin Yang, Weicheng Yang, Yu Tian

**Affiliations:** 1 School of Life Sciences, Guizhou Normal University, Guiyang, China School of Life Sciences, Guizhou Normal University Guiyang China; 2 Administration of Fanjingshan National Nature Reserve, Jiangkou, China Administration of Fanjingshan National Nature Reserve Jiangkou China

**Keywords:** Longhorn beetle, Sky Ox Beetle, taxonomy

## Abstract

**Background:**

*Anoplophora* Hope, 1839 is a genus including more than 40 species occurring in Asia. Most species of this genus have beautiful colours on the elytra and are of great interest to insect collectors. Due to their developing in and consuming wood in the larval stage, species in this genus could be economically important, such as *A. glabripennis*, an introduced species to North America.

**New information:**

We described *Anoplophora fanjingensis* sp. n., based on specimens from Mount Fanjing (also Fanjingshan), Jiankou County, Guizhou, China. The new species is characterised by its elytra with metallic iridescent sheen and the non-annulated antennae. Habitus of two similar species, *Anoplophora chiangi* and *Anoplophora leechi*, are also presented.

## Introduction

*Anoplophora* Hope, 1839 is a genus of the tribe Lamiini (Coleoptera, Cerambycidae, Lamiinae) that encompasses more than 40 species occurring in Asia (Bi and Ohbayashi 2015, Duranton 2004, Lingafelter and Hoebeke 2002, Ohbayashi and Bi 2014, Xie et al. 2012, Xie et al. 2015). Most species of this genus are characterised by: (1) dense pubescence covering the head, (2) large, conspicuous apical cicatrix on the antennal scape, (3) annuli on the antennae with pubescence of about the same colour as the elytra and/or pronotum and (4) a posteromedial callus on the pronotum (Lingafelter and Hoebeke 2002).

The aim of this study is to describe a new species of the genus *Anoplophora* from Guizhou Province, China.

## Materials and methods

A total of four specimens, including three males (one damaged) and one female, of the new species were collected by light traps in Huixiangping, Jiangkou County, Guizhou Province, China. Morphological characters were examined using an AmScope SM-4TZ stereomicroscope. Habitus pictures were taken with a Canon EOS 6D digital camera fitted with a Carl Zeiss Milvus 100 mm lenses. Male genitalia were photographed with an Olympus SZX7 stereomicroscope using an Olympus DP22 camera. Type materials were deposited in the School of Life Sciences, Guizhou Normal University, Guiyang, China (GZNULS).

### Data resources

The data underpinning the analysis reported in this paper are deposited at GBIF, the Global Biodiversity Information Facility, http://ipt.pensoft.net/resource.do?r=xxxxxx.

## Taxon treatments

### Anoplophora fanjingensis

Yang, Yang and Tian, 2020 sp. n.

831F60F7-D217-515B-9BFB-7FA3D5F33C0F

F5ADCAD9-3DA0-4DB5-BA64-970355D694E6

#### Materials

- **Type status:** Holotype. **Occurrence:** recordedBy: Boyang Li; individualCount: 1; sex: male; **Location:** country: China; stateProvince: Guizhou; county: Jiangkou; locality: Huixiangping, Mount Fanjing; verbatimElevation: ca. 1700 m; verbatimLatitude: 27°54.18’ N; verbatimLongitude: 108°42.40’ E; **Event:** year: 2017; month: 7; day: 6-14
- **Type status:** Paratype. **Occurrence:** recordedBy: Boyan Li; individualCount: 1; sex: female; **Location:** country: China; stateProvince: Guizhou; county: Jiangkou; locality: Huixiangping, Mount Fanjing; verbatimElevation: ca. 1700 m; verbatimLatitude: 27°54.18’ N; verbatimLongitude: 108°42.40’ E; **Event:** year: 2016; month: 6; day: 20
- **Type status:** Paratype. **Occurrence:** recordedBy: Boyan Li; individualCount: 1; sex: male; **Location:** country: China; stateProvince: Guizhou; county: Jiangkou; locality: Huixiangping, Mount Fanjing; verbatimElevation: ca. 1700 m; verbatimLatitude: 27°54.18’ N; verbatimLongitude: 108°42.40’ E; **Event:** year: 2016; month: 6; day: 25

#### Description

**Male**: black, body length, male (Fig. 1a) 26.4—27.4 mm (n = 2), humeral width, 9.2—9.4 mm. Elytra with purple to green metallic iridescent sheen.

Head: with sparse small punctures on the base of antennal tubercles and genae. Punctures denser on the antennal tubercles than those on the genae (Fig. 2a). Antennae exceed apex of elytra by about 5 antennomeres. Antennae coated with appressed short pale hairs. Scape cicatrix strong and with conspicuous coarse pits and sculptures. Eyes deeply emarginate, lower lobe as high as gena. Frons with a shallow middle groove that extends to the vertex. Labrum trapezoidal (Fig. 3c), wider at apex than at the base, with setae on apical half. Clypeus membranous, without setae (except for the anterior margin of frons) or apparent punctations. Maxilla (Fig. 3b) with palpi 5-segmented; apical palpomere conical; second palpomere short and strongly constricted at basal one third. Third palpomere clubbed. Fourth maxillary palpomere constricted at basal one fifth, then gradually inflated towards apex, about 1.5 times as wide at apical than at base. Labium (Fig. 3d) with palpi 4-segmented; apical palpomere football shaped, inflated at middle; penultimate palpomere inflated, about 1.6 times as wide at widest point as at base. Mandible (Fig. 3a) large, triangular, articulation prominent, cutting surface sharp, unserrated and crescent-shaped.

Thorax: pronotum black and shiny, with a weak posterior callus, but without distinct anterior and lateral calli. Anterior and posterior transverse constrictions present. Posterior transverse constriction more conspicuous than the anterior one. Glabrous dorsally, with sparse long black hairs laterally at anterior of and posterior of the pronotal spine. Coating with fine appressed hairs ventrally. With fringe of pale hairs at pronotal margin. Lateral pronotal spine strong, thickened at the base, with acute and slightly posteriorly curved apex. Procoxal cavity moderately open laterally and closed posteriorly. Mesosterna covered by numerous appressed black hairs. Scutellum glabrous, triangle-shaped with posterior apex slightly rounded (Fig. 3e). Mesepimeron contacts mesocoxa fully. Mesosternal intercoxal process covered with sparse long black hairs and with a weak ventral projection. Anterior projection not developed. Metasternum and metepisternum black, sparsely punctured and with appressed long black hairs.

Elytra: Black with purple to green iridescent sheen. Four to five small patches of indistinct pale pubescence on each elytron. Two of them on the H4 macula and I1 macula Lingafelter and Hoebeke (2002). Two small maculae on the outer margin of elytron, next to H4 and I1, respectively. The last one is small and roughly at apical 1/3 of elytron, nearly to outer margin. No erect hairs present. No granules at the base. Humeri developed. Outer margin of elytron slightly constricted at basal 1/4. Apex rounded. Sparse black hairs on the edge of elytron, denser and forming a cluster at sutural apex.

Legs: black with pubescence on tarsomeres 1—3 and tibiae. Pale pubescence on dorsal tarsomeres 1—3. Appressed short pubescence on femora, denser on the ventral side, colour of the pubescence paler. Femora cylindrical, short; metafemur extending to posterior margin of third ventrite.

Abdomen: black with coating of appressed pale setae. The apex of terminal ventrite with a shallow middle notch and a fringe of dense hairs (Fig. 5a).

Male genitalia (Fig. 4): parameres converging and touching before apices. Part of apical setae of paramere at least half length of paramere. The ventral apex of median lobe broadly rounded and tongue shaped. Median lobe slightly arcuate from lateral view. The eighth tergite transverse at base, gradually constricted towards apex. Not narrow to a point, but with a middle notch. Densely fringed at apical margin. The eighth sternite transverse, with an arcuate apical margin, with sparse pale hairs in the middle of apical half.

**Female** (Fig. 1b,Fig. 2b): body length, 31.8 mm; humeral width, 11.1 mm. Antennae with about 4 antennomeres exceeding apex of elytra, bluish hairs on the antennae, blue to green metallic iridescent sheen on the elytra, bluish pubescence on dorsal tarsomeres 1—3, weakly developed anterior projection of the mesosternal intercoxal process and middle notch at apex of terminal ventrite present (Fig. 5b).

##### Remarks

Characters of female are generally similar to male for the new species. Sexual dimorphism is exhibited in the following aspects. Body size of female is larger than body size of male. The antenna of female is with about 4 antennomeres exceeding apex of elytra (about 5 antennomeres in male). Hairs on the antennae and the dorsal tarsomeres 1—3 of female are bluish, while pale in male. Metallic iridescent sheen on the elytra of female is blue to green, while purple to green in male. Anterior projection of the mesosternal intercoxal process is weakly developed in female, but not developed in male. Middle notch at apex of terminal ventrite of female is deeper than that of male.

#### Diagnosis

*Anoplophora fanjingensis* sp. n., can be distinguished from most of its congeners by the non-annulated antennae, the pronotum with only one indistinct posterior callus and the metallic iridescent sheen on elytra. *Anoplophora chiangi* Hua & Zhang, 1991 (Fig. 1d) and *Anoplophora leechi* (Gahan, 1888) (Fig. 1c) are the most similar species to *A. fanjingensis* sp. n., but they both lack the metallic iridescent sheen on the elytra. Furthermore, *A. chiangi* has annuli on the antennae and pronounced granules at the base of the elytra. *Anoplophora leechi* lacks pubescence on the elytra. The pronotum of *A. leechi* has small calli near the base of pronotal spines while the pronotum of *Anoplophora fanjingensis* sp. n. has only an indistinct posterior callus. The new species may appear as a dark form of *Anoplophora albopicta* (Matsushita, 1933). However, *A. albopicta* was described from Taiwan and has not been recorded in mainland China (Tavakilian and Chevillotte 2019). Based on the description and images of *A. albopicta* presented by Lingafelter and Hoebeke (2002), the characters of mesonotum, scutellum and calli on the pronotum of *A. albopicta* are different from those of *A. fanjingensis* sp. n. The mesonotum of *A. fanjingensis* sp. n. (Fig. 3e) is pointed at the apical, but truncated in *A. albopicta*. The apex of the scutellum of *A. fanjingensis* sp. n. (Fig. 3e) is blunter than that of *A. albopicta*. Furthermore, middle and anterior pronotal calli are evident in *A. albopicta*, but not present in *A. fanjingensis* sp. n. This is also the case when comparing *A. fanjingensis* with *Anoplophora tonkinea* (Pic, 1907). Although the type of *A. tonkinea* is not available for examination, it was designated as *incertae sedis* by Lingafelter and Hoebeke (2002). Furthermore, according to the original description (Pic 1907), *A. tonkinea* has multiple calli on the pronotum.

#### Etymology

The specific name refers to the collecting location, Mount Fanjing.

## Supplementary Material

XML Treatment for Anoplophora fanjingensis

## Figures and Tables

**Figure 1a.**
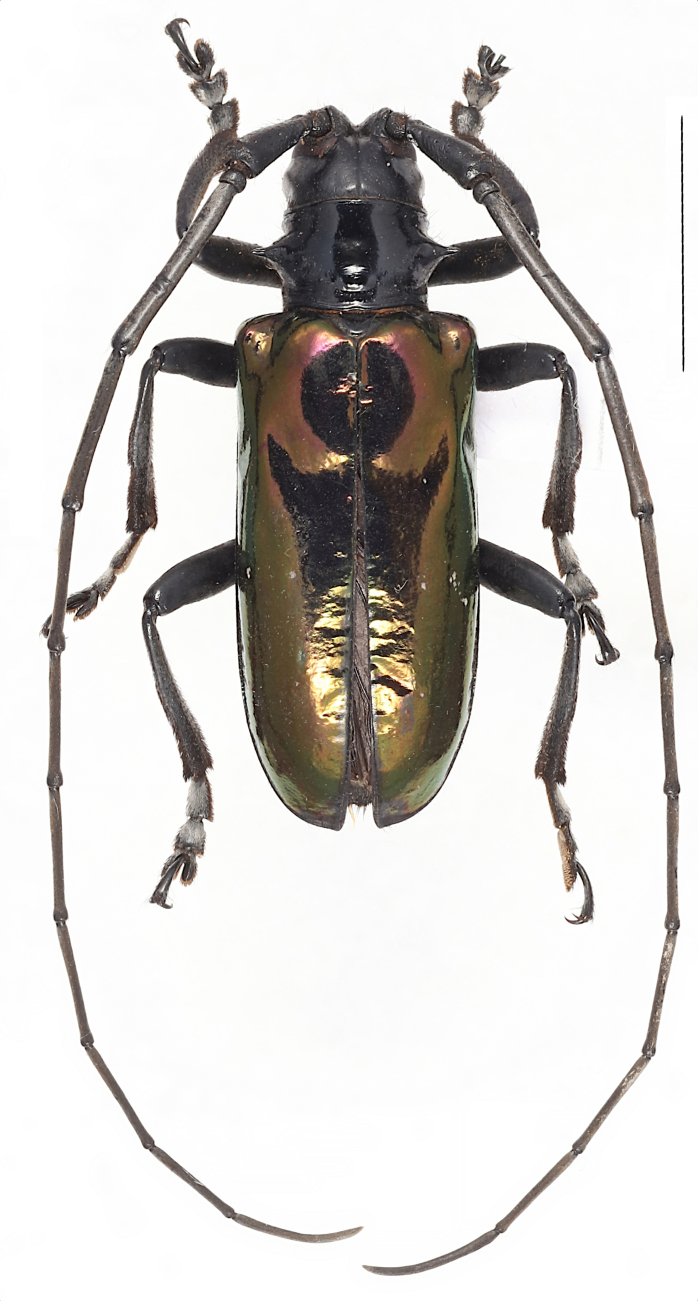


**Figure 1b.**
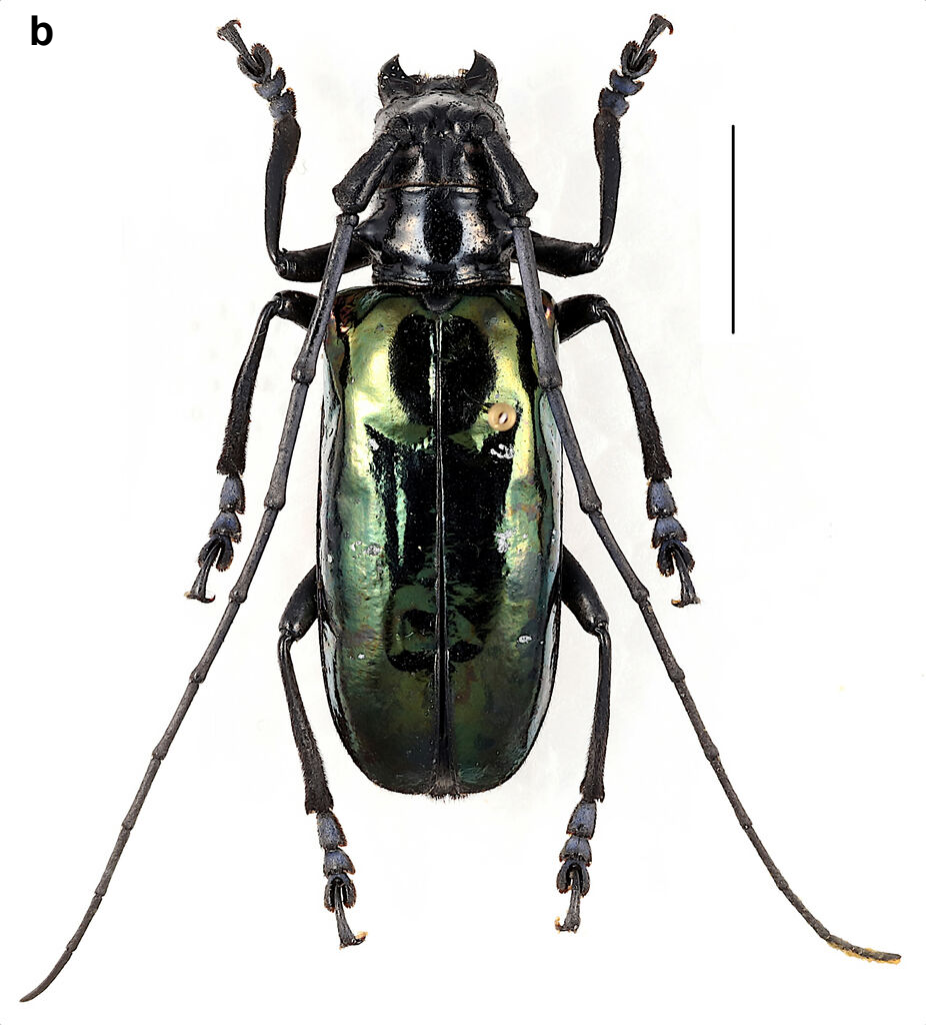


**Figure 1c.**
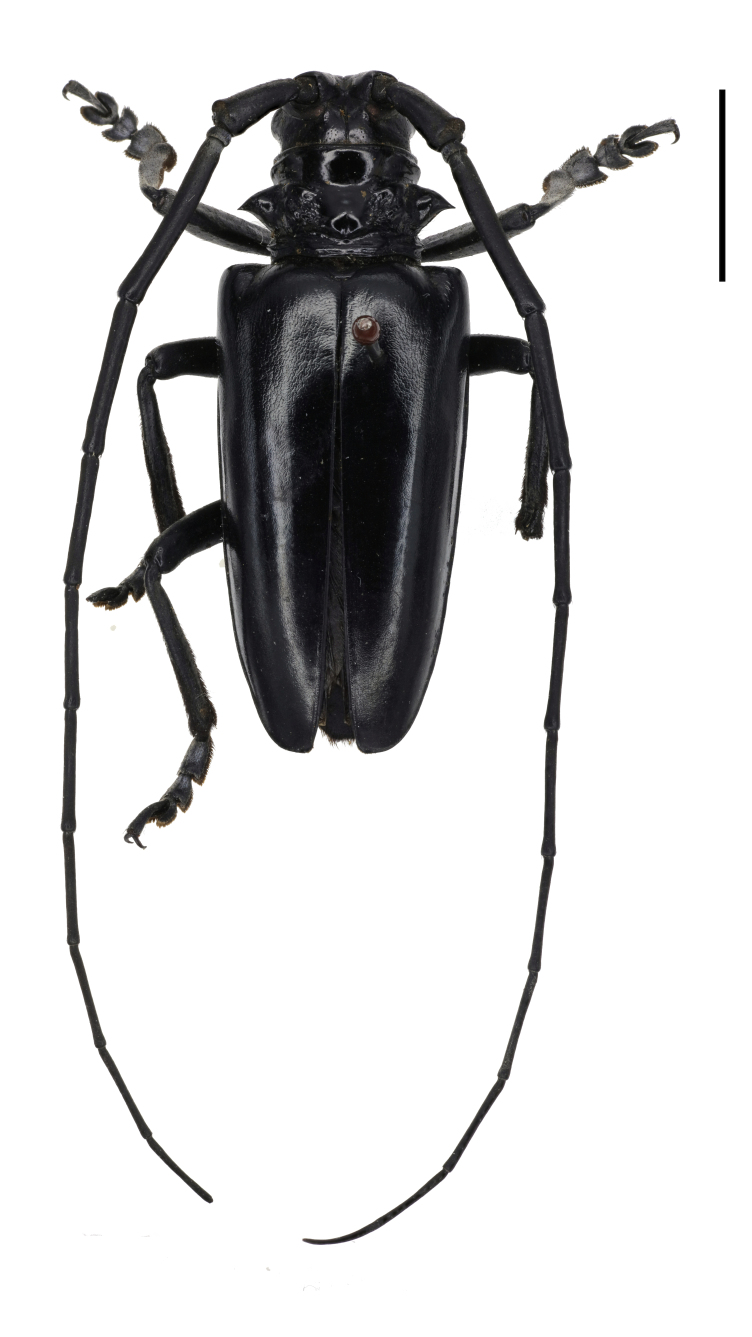


**Figure 1d.**
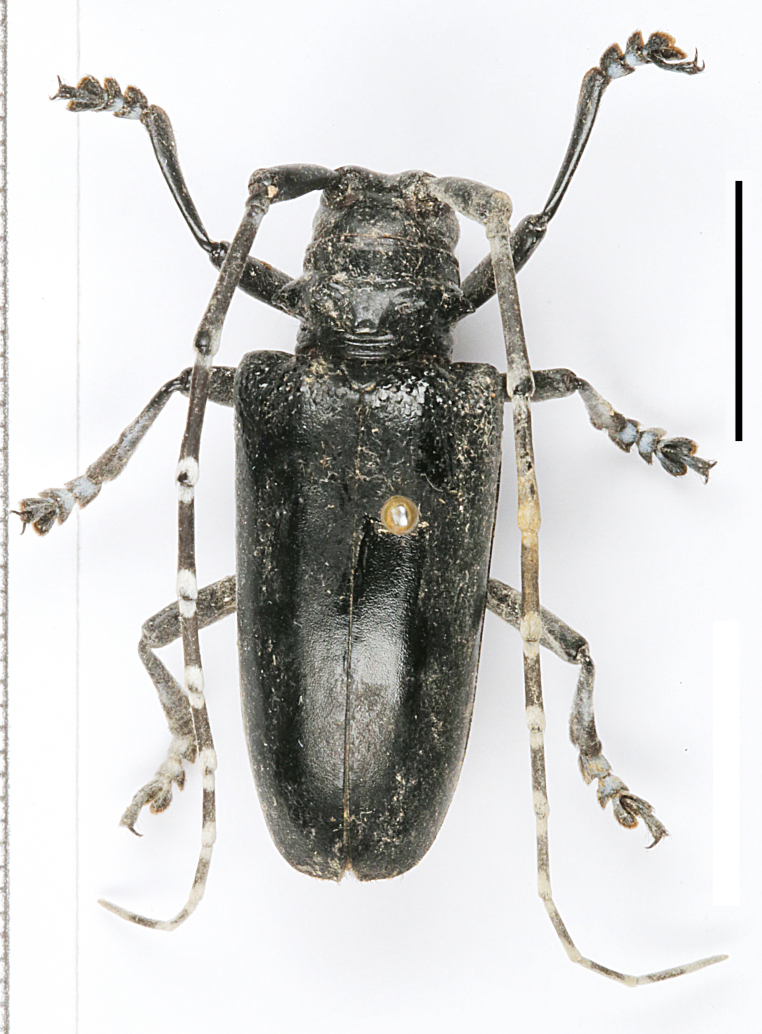


**Figure 2a.**
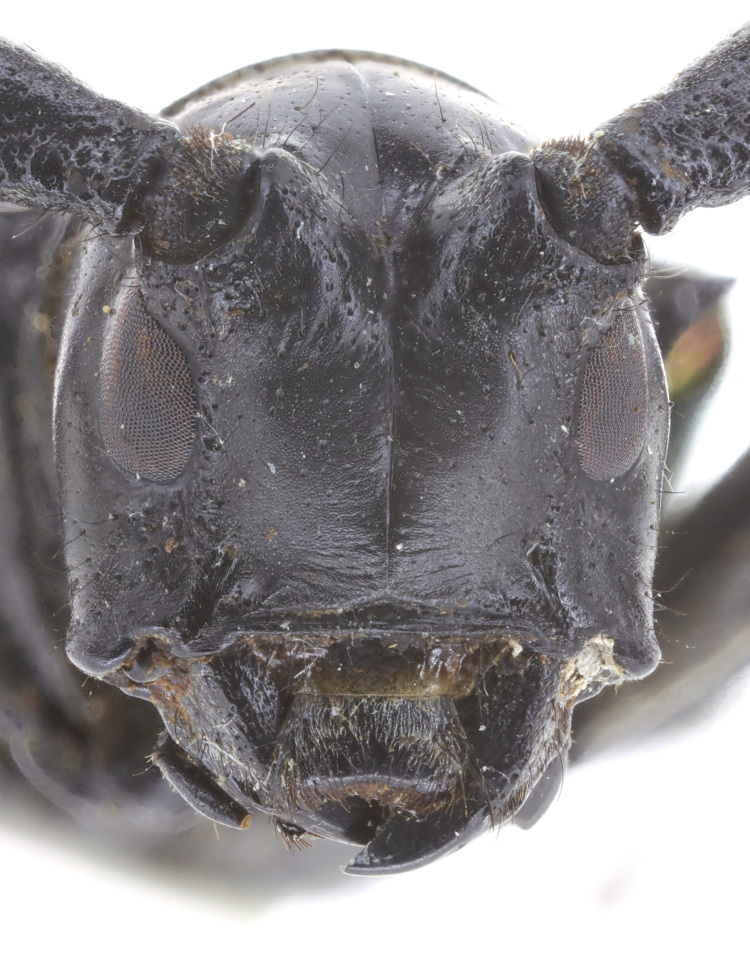


**Figure 2b.**
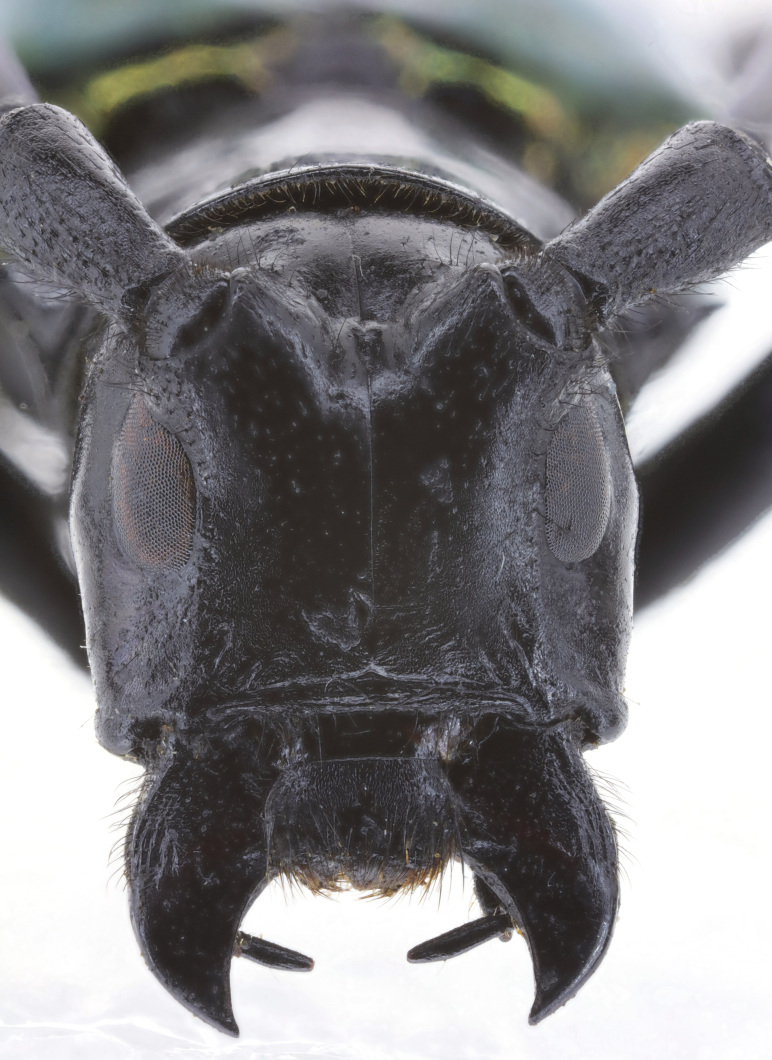


**Figure 3.**
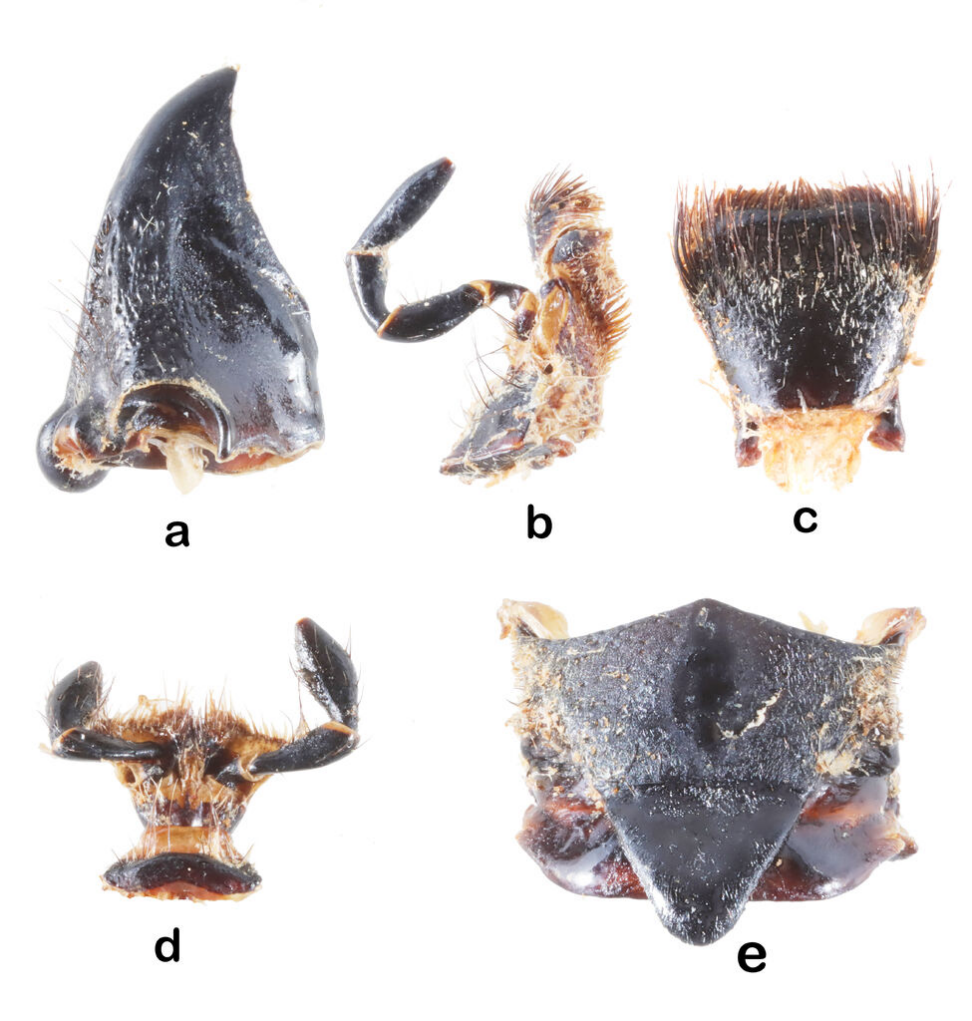
Mouthparts and mesonotum-scutellum of *A. fanjingensis* sp. n. **a.** mandible; **b.** maxilla; **c.** labrum; **d.** labium; **e.** mesonotum-scutellum.

**Figure 4.**
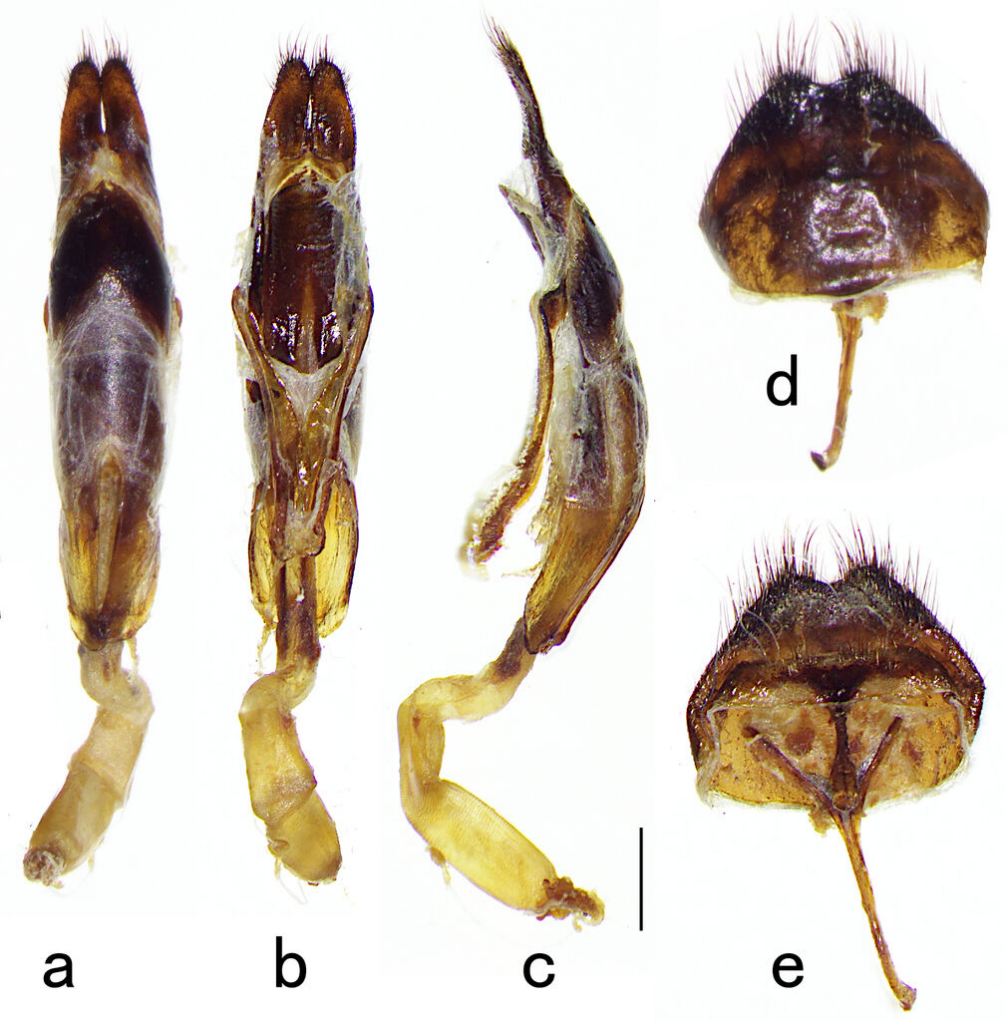
Male genitalia of *A. fanjingensis* sp. n. **a**—**c.** tegmen and median lobe; **d & e.** abdominal segment 8. (a, d, dorsal view; b, e, ventral view; c, lateral view; scale bar: 1 mm).

**Figure 5.**
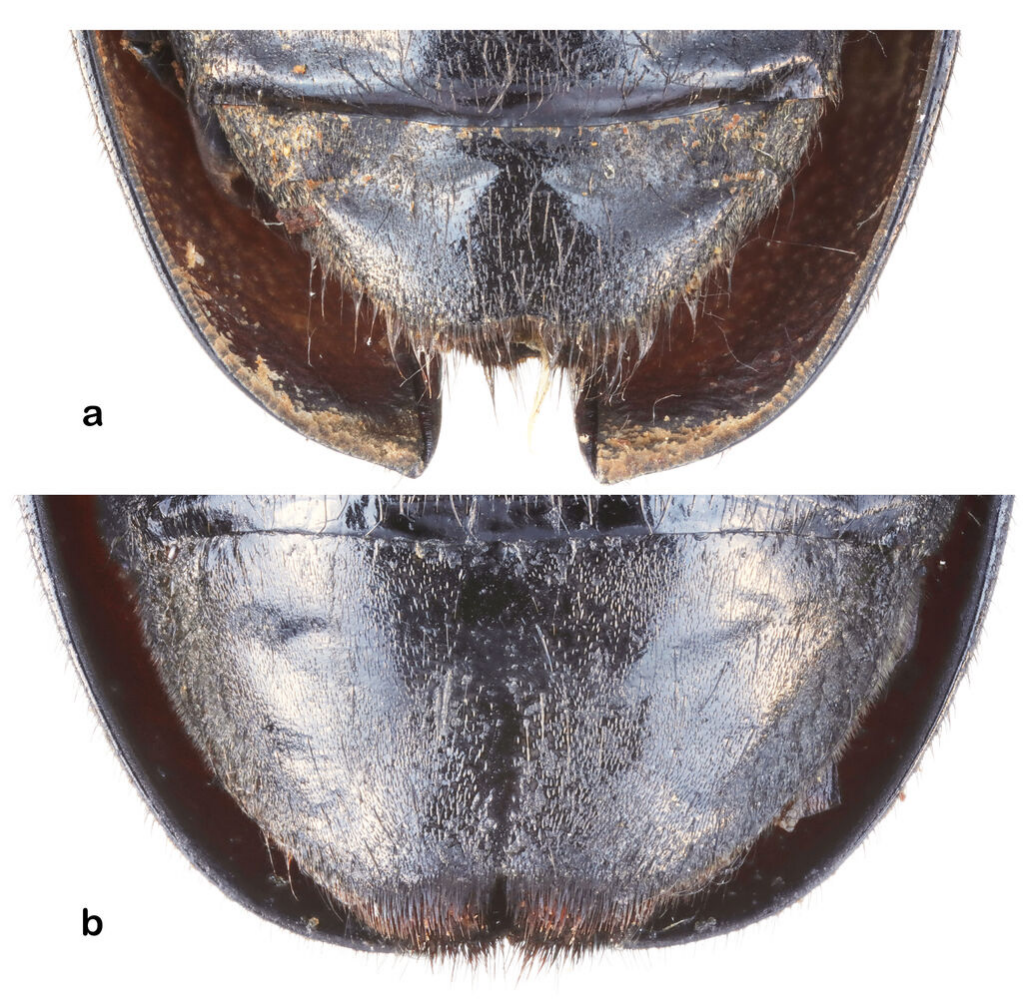
Last visable sternite of *A. fanjingensis* sp. n. **a.** male; **b.** female.
